# miR-335-5p inhibits TGF-β1-induced epithelial–mesenchymal transition in non-small cell lung cancer via ROCK1

**DOI:** 10.1186/s12931-019-1184-x

**Published:** 2019-10-21

**Authors:** Wenwen Du, Haicheng Tang, Zhe Lei, Jianjie Zhu, Yuanyuan Zeng, Zeyi Liu, Jian-an Huang

**Affiliations:** 1 Department of Respiratory Medicine, the First Affiliated Hospital of Soochow University, Suzhou, 215006 People’s Republic of China; 2 Suzhou Key Laboratory for Respiratory Diseases, Suzhou, 215006 China; 3 Department of Respiratory Medicine, The First People’s Hospital of Yancheng City, Yancheng, 224001 China; 4 Soochow University Laboratory of Cancer Molecular Genetics, Medical College of Soochow University, Suzhou, 215123 Jiangsu China; 5 Institute of Respiratory Diseases, Soochow University, Suzhou, 215006 China

**Keywords:** Non-small cell lung cancer, Metastasis, Transforming growth factor beta 1, miR-335-5p, Rho-associated protein kinase 1

## Abstract

**Background:**

Significant evidence has shown that the miRNA pathway is an important component in the downstream signaling cascades of TGF-β1 pathway. Our previous study has indicated that miR-335-5p expression was significantly down-regulated and acted as a vital player in the metastasis of non-small cell lung cancer (NSCLC), however the underlying mechanism remained unclear.

**Methods:**

The differential expression level of miR-335-5p and ROCK1 were determined by qRT-PCR and IHC analysis in human tissue samples with or without lymph node metastasis. Transwell assay was conducted to determine cell ability of migration and invasion. SiRNA interference, microRNA transfection and western blot analysis were utilized to clarify the underlying regulatory mechanism.

**Results:**

We showed that down-regulated expression of miR-335-5p and up-regulated expression of ROCK1 in NSCLC tissues were associated with lymph node metastasis. Over-expresion of miR-335-5p significantly inhibited TGF-β1-mediated NSCLC migration and invasion. Furthermore, luciferase reporter assays proved that miR-335-5p can bind to 3′-UTR of ROCK1 directly. Moreover, we confirmed that siRNA-mediated silencing of ROCK1 significantly diminished TGF-β1-mediated EMT and migratory and invasive capabilities of A549 and SPC-A1 cells.

**Conclusion:**

This is the first time to report that miR-335-5p regulates ROCK1 and impairs its functions, thereby playing a key role in TGF-β1-induced EMT and cell migration and invasion in NSCLC.

## Introduction

Non-small cell lung cancer (NSCLC) is the leading cause of cancer-related death worldwide [1, 2]. Although great progress has been made, the five-year survival rate of NSCLC patients are still below 15% [3]. It is reported that more than 90% of deaths are attributed to metastasis in solid tumors, including NSCLC [4]. Hence, in the study of the definition of the mechanism in NSCLC metastasis, a detailed understanding should be very important.

MicroRNAs (miRNAs) belong to small noncoding RNAs and the roles they play in tumor development are mediated by downstream signaling networks which can further regulate multiple cellular functions like proliferation and migration [5–8]. In particular, comprehensive expression analysis has shown that multiple miRNAs contributed to the development of lung cancer [9, 10]. Among them, miR-335-5p is mainly reported to function as a tumor suppressor of migration and invasion. In gastric cancer, miR-335-5p acts as a metastasis suppressor via targeting Bcl-w and specificity protein 1 [11]. MiR-335-5p over-expression can inhibit MCF-7 and MDA-MB-231 cell proliferation and motility partially by directly attenuating EphA4 expression in breast cancer [12]. Research in colorectal cancer indicated that patients with reduced miR-335-5p expression had a poorer overall survival and that miR-335-5p over-expression can not only inhibit tumor cells migration and invasion in vitro but also inhibit lung and liver metastasis in vivo, suggesting as a potential therapeutic target for colorectal cancer [13]. The role of miR-335-5p played in bladder cancer (BC) showed that miR-335-5p could suppress BC cell proliferation and migration by up-regulating CRKL expression [14]. However, how miR-335-5p functions in mediating tumor metastasis in NSCLC is poorly known [15, 16].

On the basis of miRNA arrays shown in our previous study, down-regulated expression of miR-335-5p is reported in NSCLC tissues when compared to adjacent tissues [17]. In another previous study, we suggested that miR-335-5p functions as a crucial regulator in the pathogenesis of NSCLC. Over-expression of miR-335-5p in A549 and H226 lung cancer cells can inhibit cell proliferation and metastasis through down-regulating CPNE1 expression, mediated via inactivation of EGFR signaling pathway [18]. Here, we predicted new potential target mRNAs of miR-335-5p using computational algorithms that may involved in metastasis of NSCLC. Interestingly, among all possible target mRNAs, we mainly focus on Rho associated protein kinase 1 (ROCK1). ROCK1 is a serine/threonine kinase protein and a direct downstream effector of Rho-GTPase signaling [19, 20]. So far the well-established role of ROCK1 supported a positive function in tumor growth. There is a growing body of evidence indicated that up-regulated expresion of ROCK1 is closely associated with β-catenin and c-myc, and thus involved in cell proliferation, cell apoptosis and cell cycle control [21, 22]. In addtion, ROCK1 can regulate tumor cell migration and invasion partially based on its role in regulating actin cytoskeleton [23, 24]. Growing evidence has also shown that the RhoA/ROCK axis can be activated as another noncanonica downstream signaling pathway in TGF-β1-induced epithelial-mesenchymal transition (EMT). It is demonstrated that LncRNA DANCR /miR-27a-3p axis can regulate EMT via ROCK1/LIMK1/COFILIN1 pathway in hepatocellular carcinoma progression [25]. In human colon adenocarcinoma, ART1 could regualte EMT process by regulating ROCK1/AKT/β-catenin pathway [26]. In NSCLC, FPPS could induce TGF-β1-induced lung cancer cell invasion and EMT via RhoA/ROCK1 pathway [27]. Interestingly, we found that ectopic miR-335-5p could inhibit TGF-β1-induced EMT and invasion of NSCLC cells.

It is well known that the transforming growth factor β1 (TGF-β1) superfamily takes crucial part in cell proliferation, apoptosis, differentiation and angiogenesis [28, 29]. As a double-edged sword, TGF-β1 inhibits cell growth in early stages [29]. In contrast, TGF-β1 can also induce EMT to promote tumor cell invasion in some degree [30]. EMT is significant for morphogenesis during the process of embryonic development and for early-stage tumors in conversion into advanced invasive malignancies, along with suppressed expression of E-cadherin and induction of N-cadherin and Vimentin [31, 32]. Accumulating evidence shows that microRNAs contribute to TGF-β1 signaling-induced EMT in various cancers. Autocrine TGF-β/ZEB /miR-200 signaling network is active and can regulate epithelial-mesenchymal transition in breast cancer [33]. In NSCLC, our previous study proved that miR-205 can moderate TGF-β1-induced EMT in A549 cell lines via repression on smad4 [34].

Our current study is to focus on elucidating the mechanism of metastasis regarding miR-335-5p in NSCLC. Data from our analysis indicated that miR-335-5p can suppress TGF-β1-induced EMT by binding to ROCK1 directly in NSCLC. Our study provided crucial evidence to illuminate the molecular and cellular mechanism of miR-335-5p-mediated suppression of metastasis, which suggested that miR-335-5p may be a promising therapeutic target for highly aggressive NSCLC.

## Materials and methods

### Patient samples

Sixty NSCLC tissue samples were collected from patients between 2012 and 2016 at the respiratory department of the First Affiliated Hospital of Soochow University. All participants were provided with written informed consent at the time of recruitment. All cases had clinically and pathologically confirmed diagnoses of NSCLC in accordance with the Revised International System for Staging Lung Cancer. Meanwhile none of these cases had received any other treatments before tissue sampling. All collected samples were further stored in a freezed condition at − 80 °C.

### ROCK1 and miR-335-5p expression in NSCLC and correlation analysis with EMT markers and overall survival

The GEPIA database (http://gepia.cancer-pku.cn/) was used to generate survival curves between ROCK1 expression and overall survival of NSCLC patients. Meanwhile we extracted data from oncoLnc database (http://www.oncolnc.org/) to validate the association between miR-335-5p expression and overall survival. To further verify the association between miR-335-3p or ROCK1 expression and EMT status in patient samples, we applied the Linkedomics database (http://www.linkedomics.org) to retrieve data on ROCK1 or miR-335-5p expression and EMT-associated marker like N-cadherin, E-cadherin, Vimentin and Snail. Meanwhile MMPs family members like MMP2 and MMP9 were also involved.

### Immunohistochemical assay

Immunohistochemical (IHC) analysis was conducted in our previou study [34]. Briefly, the sections were incubated with ROCK1 antibody (diluted to 1:100; Santa Cruze Technology, sc-17,994) overnight at 4 °C, and then incubated with the biotinylated secondary antibodies. The reactions were developed using the DAB Kit (BD Bioscience, San Jose, CA, USA), and the sections were counterstained with hematoxylin.

### Cell culture

All cell lines in this study including 16HBE, A549, HCC827, H1299, H1975, SPC-A1, H226, H1650 and H460 were obtained from the Cell bank of the Chinese Academy of Sciences (Shanghai, China) and maintained in Gibco-1640 or RPMI-1640 medium (Sigma, South Logan, UT, USA) supplemented with 10% fetal bovine serum (Gibco, South America, SA, USA) and antibiotics (Invitrogen, CA, USA) at 37 °C in a humidified incubator which contains 5% CO_2_. In some experiments, cells were cultured in special medium containing additional 5 ng/ml TGF-β1 for 48 h.

### Plasmid construction, and luciferase reporter assay

A549 and SPC-A1 cells were used for luciferase assay. In order to generate psiCHECK2-ROCK1–3′-UTR-wild type and relevant mutant plasmids, a 233 bp fragment located in the position 472–478 of ROCK1 3′- UTR which contains a potential miR-335-5p target sequence or a mutated fragment were cloned into psiCHECK2 dual luciferase vector (Promega, Madison, WI, USA). Cells were plated in 24-well plate at a optimum density of 2 × 10^4^/well and cotransfected with wild or mutant synthetic plasmid along with miR-335-5p mimics or miR-NC using jetprime reagent (Life Technologies) at the following day. 24–48 h later, cells were lysed and Dual-Luciferase Reporter Assay kit (Promega) was carried out to examine luciferase activity. Renilla luciferase activities were setted as inner control for the comparision of firefly luciferase. Each experiment was performed for three times.

### MiR-335-5p mimics, plasmid siRNA, and cell transfection

A549 and SPC-A1 cells were seeded in 6-well plates. When cells had reached 40–60% confluence, we performed transfection in accordance with the manufacturer’s instructions using jetprime reagent (Invitrogen). Cells were collected at 48–72 h after transfection for further experiments. MiR-335-5p mimics, ROCK1 siRNA (Si-ROCK1) and corresponding control were purchased from GenePharma company (Suzhou, China). The target siRNA sequences were as follows: ROCK1si-1 5′-GCAGAUGAAACAGGAAAUA-3′, ROCK1 si-2 5′-GGCAGAGGAAGAAUAUAAA-3′.

### Wound healing assay

After transfection for 48 h with miR-335-5p or si-ROCK1, A549 and SPC-A1 cells were plated into a 6-well plate with a density of 2.5 × 10^5^/well. At day 2, we gently scratched the monolayer using a 10 μl pipette tip and make sure the tip perpendicular to the bottom of the well during the process. After scratching, the wells were gently washed twice with PBS to remove the detached cells and replaced with fresh medium for an additional 12–24 h. The representative images of gap distance were acquired with microscope and quantitatively analysed and plotted with Photoshop.

### Migration and invasion assay

Transwell chambers (Corning, New York, NY, USA) were used to test the cell migration and invasion ability. For migration assay, cells were suspended and plated on uncoated chambers. The only difference between these two assays was that the inserts in invasion assay were needed to be pre-coated with diluted Matrigel matrix (BD Science, Sparks, MD, USA) at 37 °C for 2 h. For both assays, tumor cells were transfected with si-NC or si-ROCK1 for 48 h. In some conditions, cells were transfected with miR-NC or miR-335-5p mimics for 48 h, and then cultured in medium containing TGF-β1. Cells were trypsinized, and then 5 × 10^4^ cells diluted in 200 μl medium with 1% FBS were seeded into the upper champer. 800 μl RPMI-1640 medium with 10% FBS was added to the bottom of each chamber. After incubation for 24 h, non-invasive cells were removed and the remaining cells attached the inserts were fixed with methanol for 30 min, then dry for 10 min in room temperature, stained with 0.1% crystal violet overnight to make staining completely. At last we washed inserts three times with PBS. Cells were then photographed and counted. Each experiment was performed in triplicate.

### RNA extraction, cDNA synthesis, and quantitative real-time PCR analysis

RNAiso Plus reagent (Takara, Osaka, Japan) was used to extract total RNA from cells and tissues in accordance with the manufacturer′s protocol. 1 μg of total RNA was reverse-transcripted using a reverse transcriptase M-MLV kit (Takara, Osaka, Japan). mRNA expression was detected by qPCR using SYBR Master Mixture (Takara, Osaka, Japan) on an ABI Step One Plus Real-Time PCR system (Applied Biosystems, Foster City, CA, USA). Primers used for qRT-PCR were supplied by Guangzhou Ribo BioCompany (Guangzhou, China). The detailed sequences involved in this study were showed in Additional file 1 Table S1. β-actin and U6 were used as internal control. The 2^-ΔΔCt^ method was applied to figure out the relative expression. Each experiment was repeated in triplicate.

### Western blot assay

PBS was needed to be pre-cooled before experiment. After washed with PBS and 1 x RIPA lysis buffer was added to cells (Cell Signaling Technology, Danvers, MA, USA) with 1:100 phosphatase inhibitor and 1:100 protease inhibitor cocktail (Sigma-Aldrich, St. Louis, MO, USA). 10% SDS-PAGE was used to separate protein and then transferred to nitrocellulose membranes (Millipore, Billerica, MA, USA). At room temperature, membranes were blocked with 5% BSA or 5% milk for 1 h which was then dissolved in TBST buffer containing 0.1% Tween-20. Later we incubated with corresponding primary antibodies overnight at 4 °C and the appropriate secondary antibodies. After washing four times with TBST, protein detection was performed by chemiluminescence (Pierce, Rockford, IL, USA). Antibodies used in this research included anti-MMP2 (D8N9Y), anti-N-cadherin, anti-Vimentin (RV202, BD Biosciences, USA), anti-Snail (C15D3, Cell Signaling Technology, Danvers, MA, USA), and anti-mouse or rabbit secondary antibodies (Cell Signaling Technology).

### Statistical analysis

All data are presented as mean ± standard deviation (SD). *P* < 0.05 was set as statistically significant analyzed by Student’s t-test. All statistical analysis were calculated using GraphPad Prism 6.0 (GraphPad, San Diego, CA, USA) and SPSS 17.0 software (SPSS, Chicago, IL, USA).

## Results

### MiR-335-5p over-expression inhibits the migratory ability of NSCLC cells

**Fig. 1:**
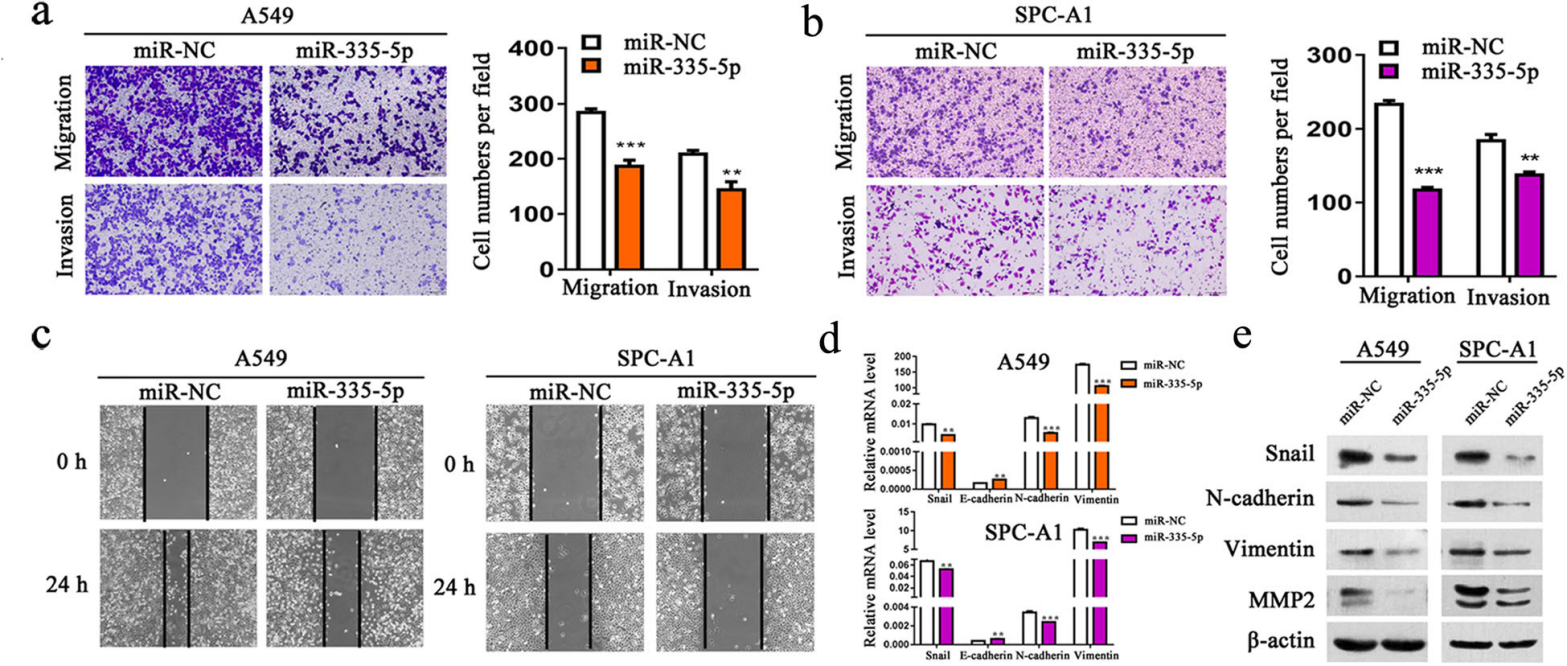
Over-expression of miR-335-5p inhibits NSCLC cell motility and migration and invasion. **a-b** Transwell assay of the cell migratory and invasive activity in A549 and SPC-A1 cell lines transfected with miR-335-5p mimics. **c** A wound-healing assay was performed to observe the effect of miR-335-5p transfection in cells. **d** The expression level of N-cadherin, Vimentin and E-cadherin by quantitative RT-PCR analysis in A549 and SPC-A1 cell lines with miR-335-5p over-expression. **e** Western blot analysis of Snail, N-cadherin, Vimentin, and MMP2 after transfected with miRNA mimics (miR-335-5p or miR-NC) for 48 h. **P < 0.01; ***P < 0.001

To evaluate the function of miR-335-5p in NSCLC, we first over-expressed miR-335-5p and then assessed the possible effects of miR-335-5p on cell migration. Results from transwell assay showed that over-expression of miR-335-5p can inhibit the migratory ability in both A549 (Fig. 1a) and SPC-A1 cells (Fig. 1b). Wound healing assay was further carried out to verify the function of miR-335-5p in regulating cell migration of A549 and SPC-A1 cells. As demonstrated in Fig. 1c, when compared with transfection with miR-NC, cells transfected with the miR-335-5p mimics lower the speed migrating towards the scratch. Consistent with these observations, qRT-PCR analysis and western blot analysis revealed decreased expression levels of Snail, N-cadherin,Vimentin and MMP2 in cells over-expressed miR-335-5p (Fig. 1d and e). In addition, no significant changes were detected regarding MMP9 and other associated transcriptional factors like Slug, ZEB1 and ZEB2 (Additional file 2 Figure S1).

### MiR-335-5p inhibits TGF-β1-induced EMT, cell migration and invasion of NSCLC cells

**Fig. 2:**
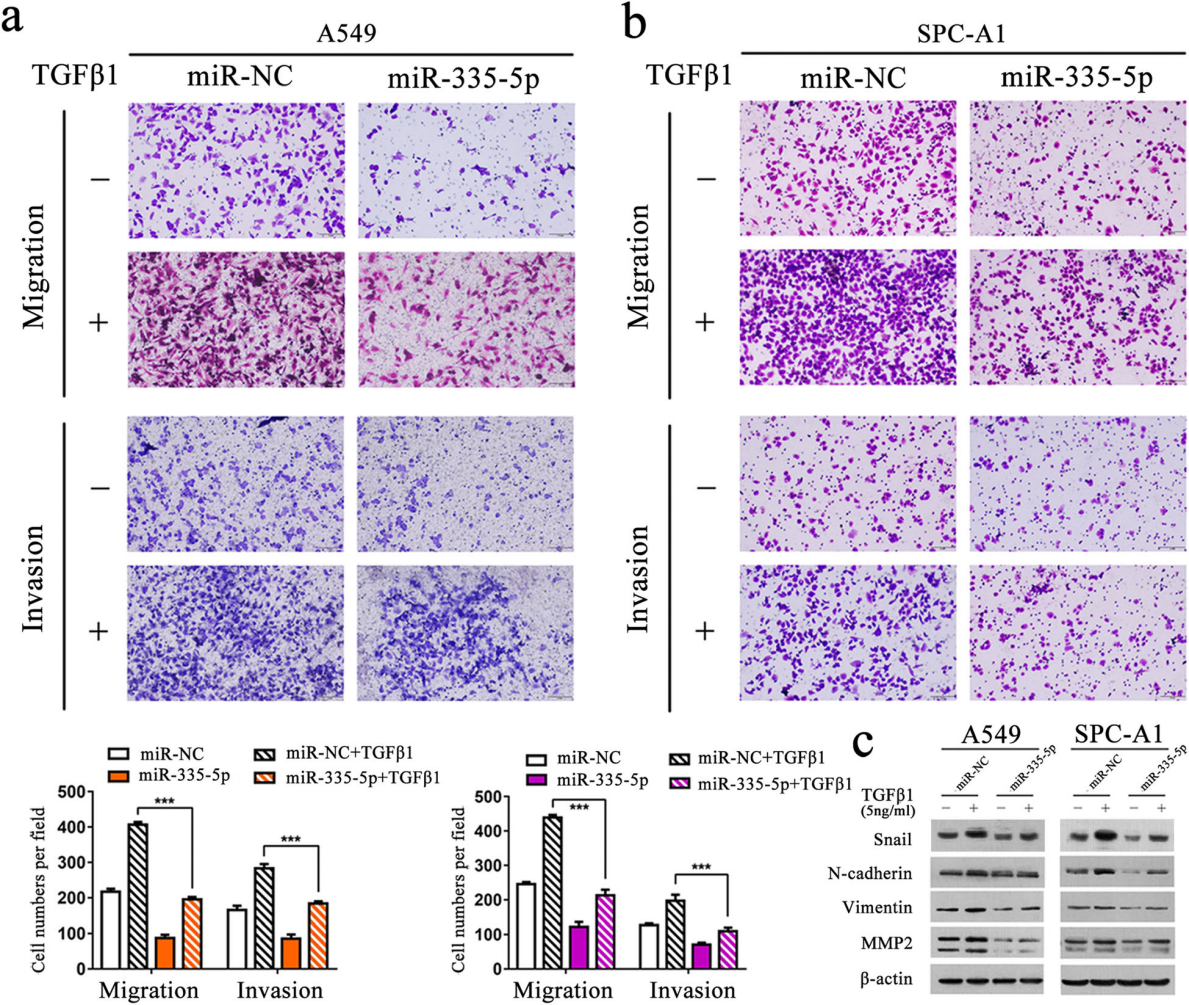
Over-expression of miR-335-5p significantly inhibits TGF-β1-induced EMT and cell migration and invasion of NSCLC cells. **a-b** Transwell assay of A549 and SPC-A1 cells after transfected with miR-335-5p or miR-NC for 24 h, then cells were serum starved for 24 h and treated with or without TGF-β1 (5 ng/ml) for 24 h. **c** Western blot analysis of the expression of Snail, N-cadherin, Vimentin and MMP2 in A549 and SPC-A1 cells transfected with miR-335-5p mimics or miR-NC in the absence or presence TGF-β1 (5 ng/ml). ***P < 0.001

In next step, the transwell assays were performed to clarify how miR-335-5p functions to induce cell invasion mediated by TGF-β1. We found that the TGF-β1-induced increase of migration and invasion abilities were inhibited after transfection with miR-335-5p in A549 or SPC-A1 cells (Fig. 2a and b). Furthermore, we performed western blot analysis to deeply clarify the role of miR-335-5p in TGF-β1-induced EMT in NSCLC cells. As shown in Fig. 2c, miR-335-5p over-expression significantly inhibited the TGF-β1-induced up-regulation of Snail, N-cadherin, Vimentin and MMP2 in both A549 and SPC-A1 cells. Our results suggested that miR-335-5p inhibited TGF-β1-induced migration and invasion in NSCLC cells.

### MiR-335-5p targets ROCK1 via 3′-UTR-binding in NSCLC cells

**Fig. 3:**
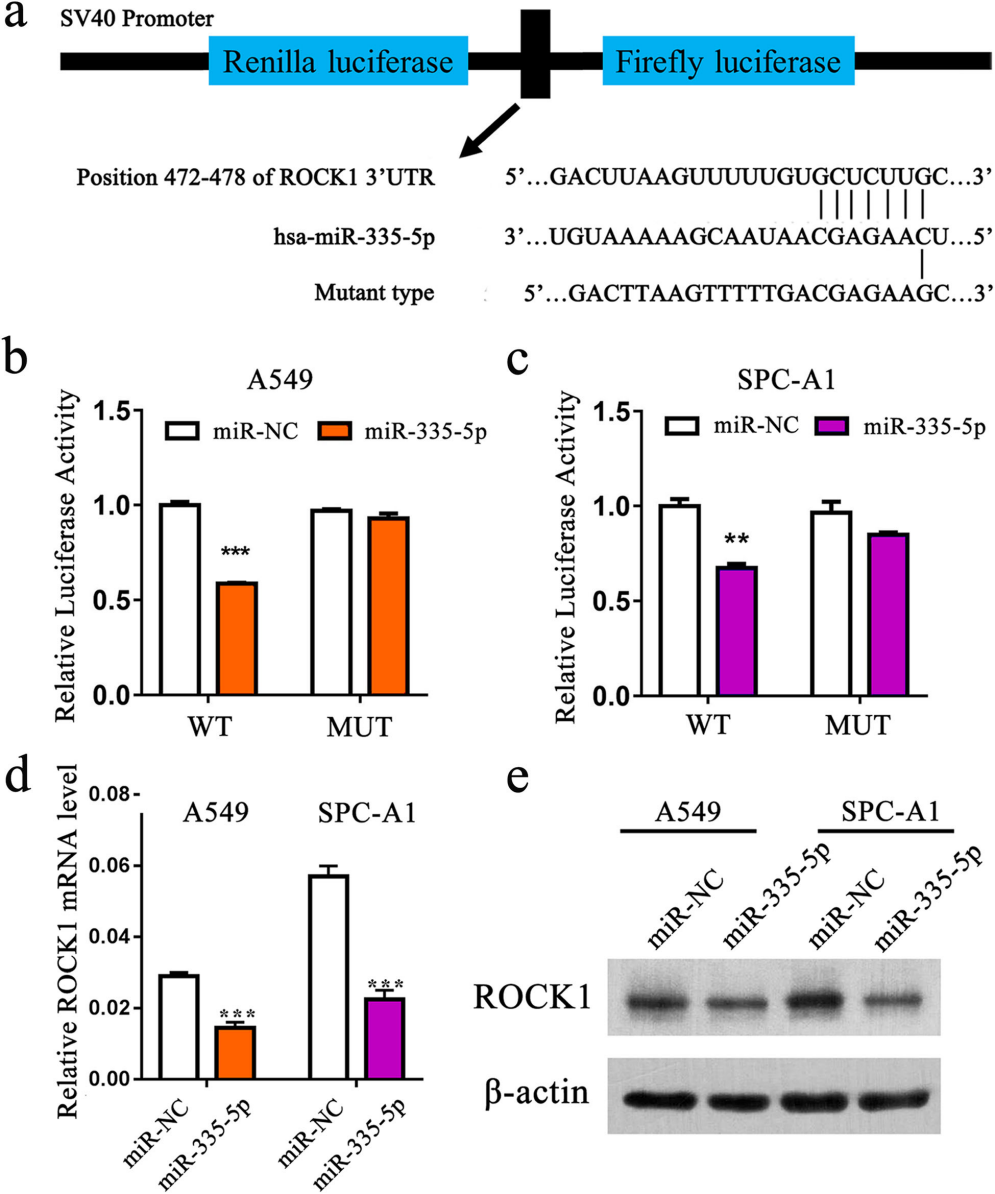
ROCK1 is a direct target of miR-335-5p. **a** Schematic diagram showing the subcloning of the predicted miR-335-5p binding site (position 472–478) of the ROCK1 3′-UTR into a psiCHECK-2 luciferase construct. Predicted duplex formation between miR-335-5p and the wild type or mutant miR-335-5p binding site is indicated. **b-c** Luciferase activity of the constructs containing the wild type or mutant ROCK1 3′-UTR reporter gene in A549 and SPC-A1 cells co-transfected with miR-NC or miR-335-5p. Relative Renilla luciferase activity was determined and normalized against the firefly luciferase activity. **d-e** qRT-PCR and western blot analysis of the expression of ROCK1 in cells transfected with miR-335-5p mimics. **P < 0.01; ***P < 0.001

To explore the mechanism by which miR-335-5p regulates NSCLC cell progression, we searched for potential regulatory targets of miR-335-5p on the basis of bioinformatics methods. Using publicly available databases (Target Scan Human: http://www.targetsca n.org/), we identified that miR-335-5p can bind to the complementary sequence of 3′-UTR of ROCK1 (Fig. 3a). To validate this hypothesis, ROCK1 wildtype and mutated reporter plasmids were generated and A549 and SPC-A1 cells were used to perform dual-luciferase reporter assays. The data manifested that the luciferase activity was largely inhibited with miR-335-5p over-expresssion when cells transfected with wildtype construct but did not with the mutant construct (Fig. 3b and c). In addition, qRT-PCR and western blot analysis exhibited that ROCK1 expression in A549 and SPC-A1 cells was detected to be reduced after transfection with miR-335-5p mimics (Fig. 3d and e). Collectively, our data certified that miR-335-5p can target 3′-UTR of ROCK1 mRNA directly in NSCLC cells.

### Reduced miR-335-5p expression and increased ROCK1 expression are associated with lymph node metastasis in NSCLC tissues

**Fig. 4:**
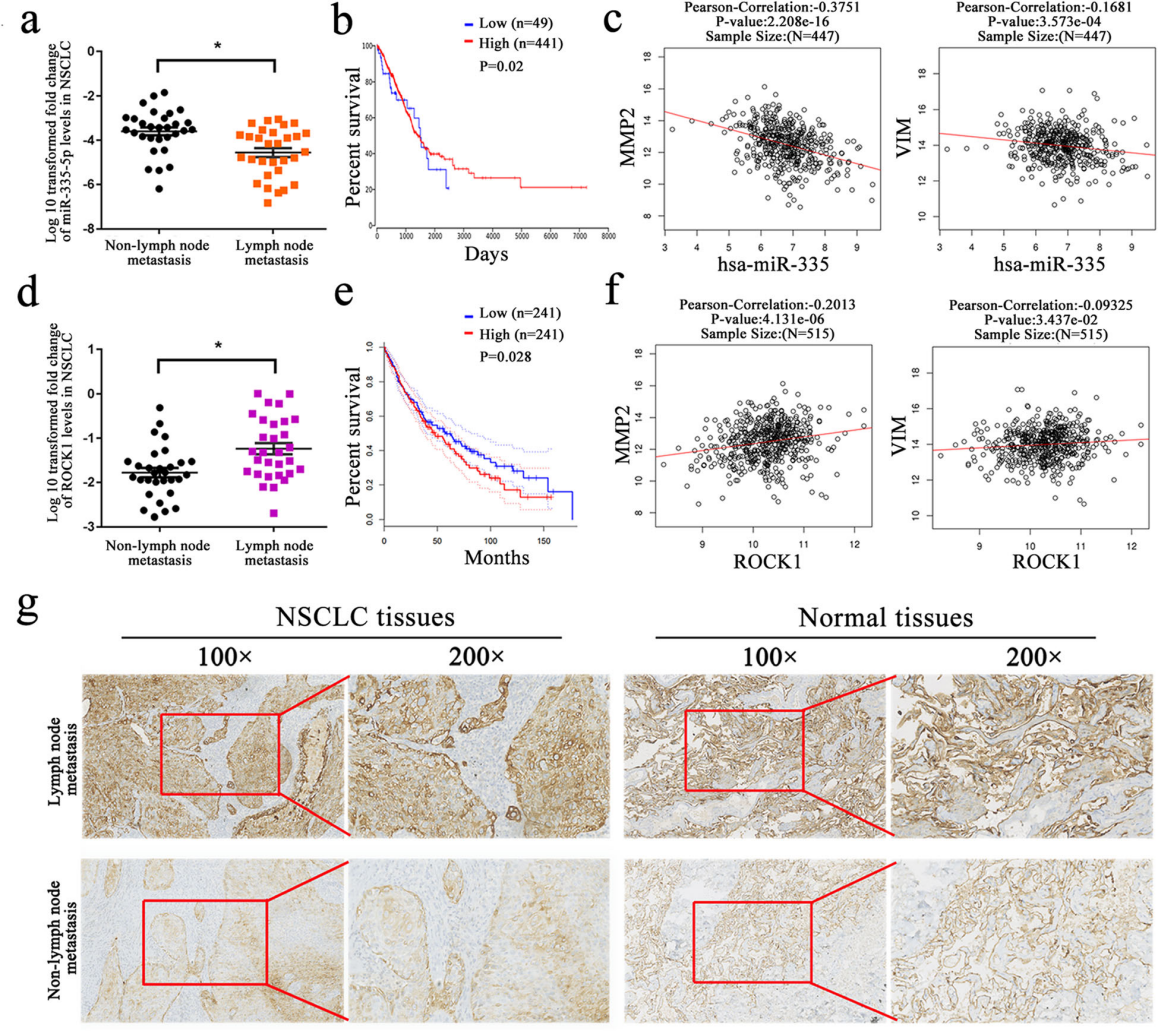
Reduced miR-335-5p expression and increased ROCK1 expression are associated with lymph node metastasis in NSCLC tissues. **a**, **d** Reduced miR-335-5p expression and increased ROCK1 expression were associated with lymph node metastasis in NSCLC tissues when compared to NSCLC tissues without metastasis. **b**, **e** Kaplan-Meier analysis showed that patients with higher miR-195-5p expression and lower ROCK1 expression were associated with favorable overall survival. **c**, **f** MiR-335-5p expression was negatively related with MMP2 and Vimentin expression in 447 patient samples. And the expression of ROCK1 is positively associated with MMP2 and Vimentin expression in 515 patient samples. **g** IHC analysis of ROCK1 expression in lymph-node matastasis and non-lymph node metastasis group. *P < 0.05

The expression level of miR-335-5p and ROCK1 in 60 paired NSCLC tissues were further classified to subgroups according to presence or absence of lymph node metastasis (Additional file 3 Table S2). Reduced miR-335-5p expression was associated with lymph node metastasis in NSCLC tissues when compared to NSCLC tissues without metastasis (Fig. 4a). Meanwhile, based on data extracted form GEPIA and oncoLnc database, we further explored that patients with higher miR-335-5p expression was associated with more favorable overall survival (Fig. 4b). In particular, we found that miR-335-5p expression was negatively related with MMP2 and Vimentin expression in 447 patient samples from linkedomics database (Fig. 4c). In addition to miR-335-5p, up-regulated ROCK1 expression was found in NSCLC tissues with lymph node metastasis, displaying poorer overall survival (Fig. 4d and e). And linkedomics database indicatd that ROCK1 expression was positively related with MMP2 and Vimentin expression in 515 patient samples (Fig. 4f). Moreover, we performed IHC analysis to compare ROCK1 expression in 10 patients samples with or without lymph node metastasis seperately. The data demonstrated that ROCK1 was mainly located in cell cytoplasm and membrane. In lymph node metastasis group, ROCK1 expression was found to be higher in 6 NSCLC tissues out of 10 NSCLC tissues. However, in 10 non-lymph node meatstasis tissues, only 2 tissues showed high ROCK1 expression. And the representative images were shown in Fig. 4g.

### Knockdown of ROCK1 inhibits TGF-β1-induced EMT, cell migration and invasion of NSCLC cells

**Fig. 5:**
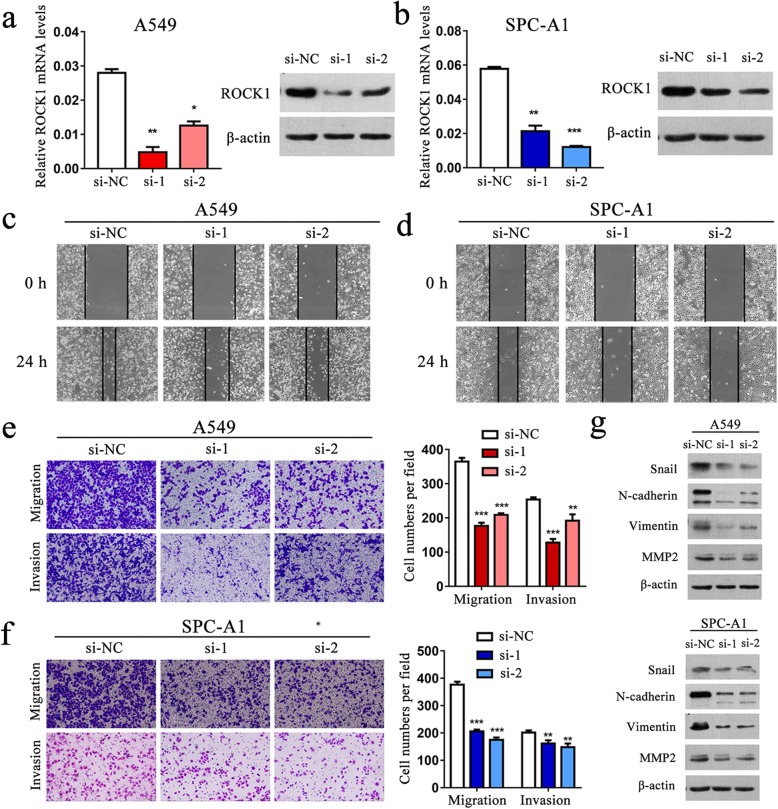
Knockdown of ROCK1 inhibits the migratory and invasive ability in A549 and SPC-A1 cells. **a-b**. qRT-PCR and western blot analysis of ROCK1 expression level in A549 and SPC-A1 cells after transiently transfected with specific siRNA (si-ROCK1) or negative control (si-NC). **c-d**. The wound healing assay of cells after silencing ROCK1 expression. **e-f**. The transwell assay showed that knockdown of ROCK1 can inhibit cell migration and invasion in A549 and SPC-A1 cells. **g**. Western blot analysis of Snail, N-cadherin, Vimentin and MMP2 expression in A549 and SPC-A1 cells after ROCK1 knockdown. *P < 0.05; **P < 0.01; ***P < 0.001

Multiple studies indicated that ROCK1 functions as an oncogene, and that ROCK1 over-expression promotes invasion and metastasis in lung cancer cells [27]. However, the role of ROCK1 has not been fully explored in TGF-β1-induced EMT, especially in lung cancer. To address this, we first inhibited ROCK1 using two specific small interfering RNAs (siRNAs) and found that ROCK1 expression was significantly reduced in A549 and SPC-A1 cells (Fig. 5a and b). In wound healing assays, cells transfected with si-ROCK1 migrated towards the scratch more slowly (Fig. 5c and d). Transwell assay showed similar results (Fig. 5e and f). Moreover, western blot revealed decreased protein levels of Snail, N-cadherin, Vimentin and MMP2 in cells with ROCK1 knockdown (Fig. 5g).

**Fig. 6:**
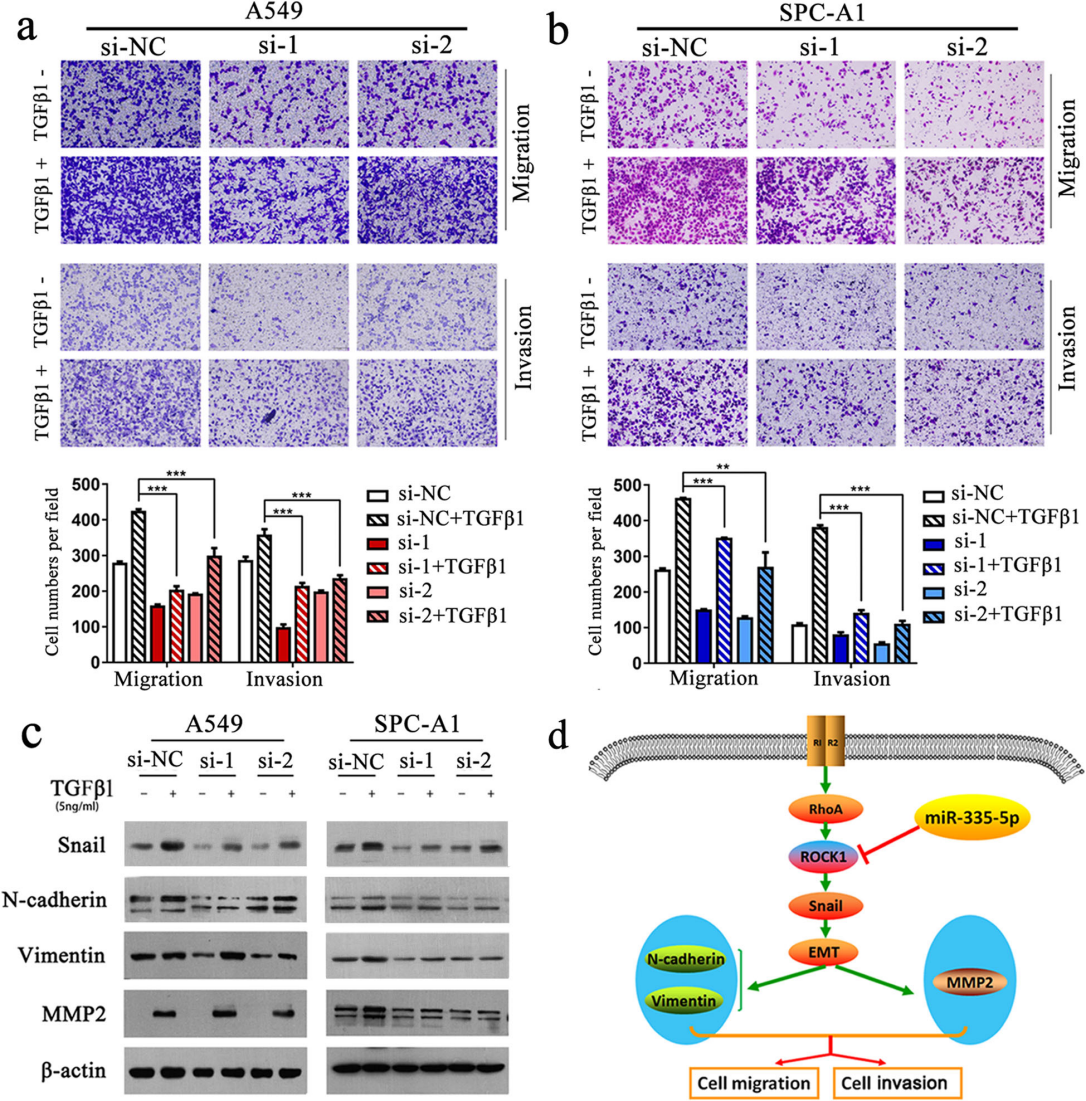
Knockdown of ROCK1 significantly inhibits TGF-β-induced EMT and cell migration and invasion of NSCLC cells. **a-b**. Transwell assay of the ROCK1-silenced A549 and SPC-A1 cells after treated with or without TGF-β1 (5 ng/ml) for 24 h. **c**. Western blot analysis of Snail, N-cadherin, Vimentin, and MMP2 expression in A549 and SPC-A1 cells after siRNA (si-ROCK1 or si-NC) transfection for 24 h and treated with exogenous TGF-β1 (5 ng/ml) for additional 24 h. **d**. A working model of the mechanistic interaction of miR-335-5p and ROCK1 in the control of TGF-β1-induced cell migration and invasion. ***P < 0.001

Next, the role ROCK1 played in promoting metastasis of NSCLC cells induced by TGF-β1 was evaluated. In the presence of TGF-β1, knockdown of ROCK1 can obviously diminished EMT and the migration and invasion of A549 and SPC-A1 cells (Fig. 6a and b). Consistent with these observations, western blot analysis confirmed that knockdown of ROCK1 noticeably inhibited the TGF-β1-induced up-regulated expression of Snail, N-cadherin, Vimentin and MMP2 in A549 and SPC-A1 cells (Fig. 6c).

## Discussion

A majority of lung cancer patients are already at an advanced stage of tumor progression when diagnosed at the first time [3]. One of the most studied mechanisms of tumor metastasis is TGF-β1-mediated EMT, which is associated with the conversion of early-stage tumors into invasive malignancies [14]. TGF-β1-mediated signal transduction functions to promote EMT in many cancers like lung cancer [30]. TGF-β1-induced EMT in malignant cells is characterized as suppressed expression of E-cadherin along with increased expression of N-cadherin and Vimentin [34].

It is well known that TGF-β1 activates multiple intracellular signaling molecules to mediate cellular functions indepedent of Smads. RhoA/ROCK family function as downstream effectors in the TGF-β1-induced non-Smad signaling pathways [35, 36]. ROCK1 and ROCK2 are two isoforms in ROCK family and display similarities in their kinase activity domains [37]. Human ROCK1 locates in chromosome 18 (18q11.1) [38]. ROCK1 is proved to function as a positive regulator in promoting invasion and metastasis in many types of solid tumors, including bladder [39], hepatocellular carcinoma [40], breast cancer [41] and lung cancers [42, 43].

In this study, we first elucidated how miR-335-5p regulated NSCLC metastasis. EMT can be induced by TGF-β1 signal in NSCLC [34], and a study reported that miRNA-335-5p can bind to genes involved in non-canonical TGF-β1 signalling pathway to suppress cell invasion [44]. We verified the impact of miR-335-5p on EMT induced by TGF-β1 in NSCLC cells. Our data demonstrated that NSCLC cells over-expressing miR-335-5p showed decreased migration and invasion abilities. Meanwhile siRNA-mediated silencing of ROCK1 distinctly diminished EMT induced by TGF-β1 and the migration and invasion in A549 and SPC-A1 cells. Further, we revealed that down-regulated expression of miR-335-5p and up-regulated expression of ROCK1 in NSCLC tissues were closely relevant to lymph node metastasis. Taken together, our data suggested that miR-335-5p over-expression can negatively regulate the expression of ROCK1 and TGF-β1-induced EMT and cell migration and invasion in NSCLC cells.

## Conclusion

In conclusion, our study is the first to report that miR-335-5p regulates ROCK1 and impairs its functions, thereby playing a key role in TGF-β1-induced EMT and cell migration and invasion in NSCLC (Fig. 6d). Our findings illuminate the inner binding interaction between ROCK1 and miR-335-5p in NSCLC metastasis, and underscore the significance of miR-335-5p as a novel target for therapeutic intervention in advanced NSCLC.

## Supplementary information

**Additional file 1: Table S1.** The sequences of primers involved in our study.

**Additional file 2: Figure S1.** The expression of MMP9 and associated transcriptional factors Slug, ZEB1 and ZEB2 after transfected with miR-335-5p mimics in A549 and SPC-A1 cells.

**Additional file 3: Table S2.** Clinicopathological features of NSCLC tissues from 30 patients with non-lymph node metastasis and 30 patients with lymph node metastasi.

## Data Availability

The original source data and material will be available upon resonable request. And the Kaplan-Meier survival curves were obtained from GEPIA database (http://gepia.cancer-pku.cn/) and oncoLnc database (http://www.oncolnc.org/). The association analysis is available in the Linkedomics database (http://www.linkedomics.org).

## Abbreviations

3′-UTR: 3′-Untranslated region
EMT: Epithelial–mesenchymal transition
IHC: Immunohistochemistry
miR-335-5p: microRNA-335-5p
miRNA: microRNA
NC: Negative control
NSCLC: Non-small cell lung cancer
ROCK1: Rho associated protein kinase 1
RT-PCR: Real-time polymerase chain reaction
TGF-β1: Transforming growth factor-β1
