# Supplementary material for: miR-335-5p inhibits TGF-β1-induced epithelial–mesenchymal transition in non-small cell lung cancer via ROCK1
Source: Respir Res. 2019 Oct 21;20:225. doi: 10.1186/s12931-019-1184-x (PMC6805547; doi:10.1186/s12931-019-1184-x)
Supplement: Supplementary file 1 — Additional file 1: Table S1. The sequences of primers involved in our study. [file 12931_2019_1184_MOESM1_ESM.docx]

| **Nane** | **position** | **Sequences (**5'‑3'**)** |
| --- | --- | --- |
| ROCK1 | FP | AACATGCTGCTGGATAAATCTGG |
|  | RP | TGTATCACATCGTACCATGCCT |
| Snail | FP | CGAAAGGCCTTCAACTGCAAAT |
|  | RP | ACTGGTACTTCTTGACATCTG |
| Slug | FP | TGTTGCAGTGAGGGCAAGAA |
|  | RP | GACCCTGGTTGCTTCAAGGA |
| ZEB1 | FP | TTCAAACCCATAGTGGTTGCT |
|  | RP | TGGGAGCACCAAACCAACTG |
| ZEB2 | FP | ACTTTTCCTGCCCTCTCTGT |
|  | RP | TTGCGATTACCTGCTCCTT |
| E-cadherin | FP | CGAAAGGCCTTCAACTGCAAAT |
|  | RP | ACTGGTACTTCTTGACATCTG |
| N-cadherin | FP | TGGGAATCCGACGAATGG |
|  | RP | TGCAGATCGGACCGGATACT |
| Vimentin | FP | TGAGTACCGGAGACAGGTGCAG |
|  | RP | TAGCAGCTTCAACGGCAAAGTTC |
| β-actin | FP | CACAGAGCCTCGCCTTTGCC' |
|  | RP | ACCCATGCCCACCATCACG |

**Table S1. The sequences of primers involved in our study**
