# Supplementary figures and images for: miR-335-5p inhibits TGF-β1-induced epithelial–mesenchymal transition in non-small cell lung cancer via ROCK1
Source: Respir Res. 2019 Oct 21;20:225. doi: 10.1186/s12931-019-1184-x (PMC6805547; doi:10.1186/s12931-019-1184-x)

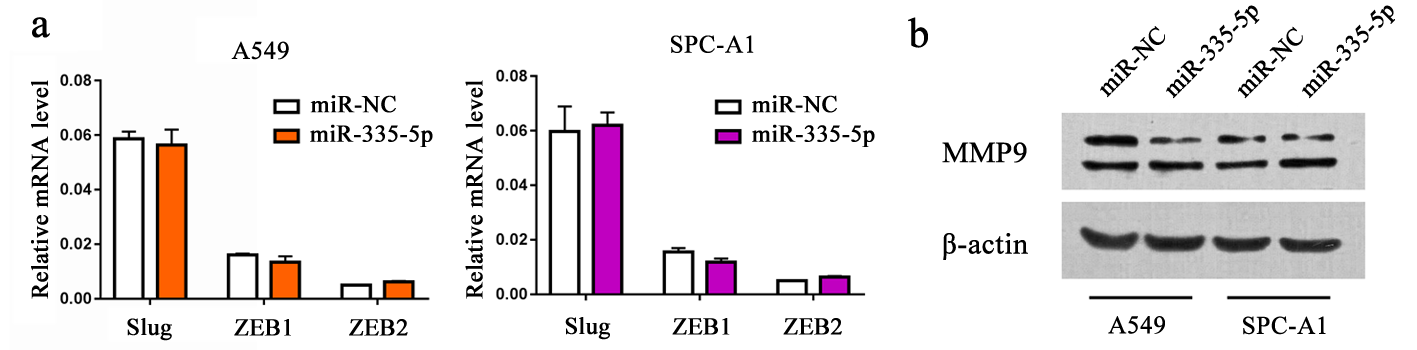

Supplement: Supplementary file 2 — Additional file 2: Figure S1. The expression of MMP9 and associated transcriptional factors Slug, ZEB1 and ZEB2 after transfected with miR-335-5p mimics in A549 and SPC-A1 cells. [file 12931_2019_1184_MOESM2_ESM.tif]
