# Supplementary material for: miR-335-5p inhibits TGF-β1-induced epithelial–mesenchymal transition in non-small cell lung cancer via ROCK1
Source: Respir Res. 2019 Oct 21;20:225. doi: 10.1186/s12931-019-1184-x (PMC6805547; doi:10.1186/s12931-019-1184-x)
Supplement: Supplementary file 3 — Additional file 3: Table S2. Clinicopathological features of NSCLC tissues from 30 patients with non-lymph node metastasis and 30 patients with lymph node metastasi. [file 12931_2019_1184_MOESM3_ESM.doc]

| **Table S2. Clinicopathological features of NSCLC tissues from 30 patients with** | | | | | | | | | | |
| --- | --- | --- | --- | --- | --- | --- | --- | --- | --- | --- |
| **non-lymph node metastasis and 30 patients with lymph node metastasis** | | | | | | | | | | |
| Non-lymph node metastasis | | | | |  | Lymph node metastasis | | | | |
| Case | Age | Stage | TNM | Histology |  | Case | Age | Stage | TNM | Histology |
| 1 | 54 | IA | T1N0M0 | AdC |  | 1 | 70 | IIIB | T3N1M0 | SqC |
| 2 | 64 | IA | T1N0M0 | SqC |  | 2 | 65 | IIIA | T2N2M0 | AdC |
| 3 | 64 | IIA | T2N0M0 | SqC |  | 3 | 65 | IIIA | T2N1M0 | AdC |
| 4 | 62 | IIA | T2N0M0 | AdC |  | 4 | 35 | IIIA | T2N2M0 | AdC |
| 5 | 65 | IA | T1N0M0 | AdC |  | 5 | 72 | IIIB | T4N2M0 | SqC |
| 6 | 72 | IIA | T2N0M0 | AdC |  | 6 | 69 | IIIA | T2N2M0 | AdC |
| 7 | 58 | IIB | T3N0M0 | AdC |  | 7 | 62 | IIIA | T2N2M0 | Other |
| 8 | 87 | IB | T2N0M0 | AdC |  | 8 | 72 | IIIA | T2N2M0 | SqC |
| 9 | 53 | IA | T1N0M0 | AdC |  | 9 | 64 | IIIA | T2N2M0 | SqC |
| 10 | 54 | IA | T1N0M0 | AdC |  | 10 | 55 | IIIB | T1N2M0 | SqC |
| 11 | 72 | IIA | T2N0M0 | AdC |  | 11 | 62 | IIIA | T2N1M0 | AdC |
| 12 | 71 | IIA | T2N0M0 | AdC |  | 12 | 59 | IIIA | T3N2M0 | Other |
| 13 | 66 | IIA | T2N0M0 | AdC |  | 13 | 62 | IIIA | T2N2M0 | SqC |
| 14 | 67 | IA | T1N0M0 | AdC |  | 14 | 75 | IIIA | T2N2M0 | Other |
| 15 | 65 | IA | T1N0M0 | SqC |  | 15 | 32 | IV | T2N2M1 | Other |
| 16 | 67 | IB | T2N0M0 | AdC |  | 16 | 60 | IIIA | T2N2M0 | AdC |
| 17 | 52 | IB | T2N0M0 | AdC |  | 17 | 62 | IIIA | T2N2M0 | AdC |
| 18 | 52 | IB | T2N0M0 | AdC |  | 18 | 64 | IIIA | T3N2M0 | Other |
| 19 | 65 | IA | T1N0M0 | AdC |  | 19 | 61 | IIIA | T2N2M0 | Other |
| 20 | 71 | IB | T2N0M0 | AdC |  | 20 | 59 | IIIB | T4N2M0 | AdC |
| 21 | 73 | IA | T1N0M0 | Other |  | 21 | 49 | IIIA | T2N2M0 | AdC |
| 22 | 67 | IIA | T2N0M0 | SqC |  | 22 | 59 | IIIB | T2N2M0 | AdC |
| 23 | 65 | IA | T1N0M0 | AdC |  | 23 | 78 | IIIA | T4N1M0 | AdC |
| 24 | 64 | IA | T1N0M0 | Other |  | 24 | 74 | IIIA | T2N2M0 | SqC |
| 25 | 77 | IIA | T2N0M0 | Other |  | 25 | 65 | IIIA | T2N2M0 | Other |
| 26 | 57 | IB | T2N0M0 | AdC |  | 26 | 55 | IIIB | T2N2M0 | AdC |
| 27 | 61 | IA | T1N0M0 | SqC |  | 27 | 64 | IV | T3N2M1 | AdC |
| 28 | 72 | IB | T2N0M0 | SqC |  | 28 | 56 | IIIA | T2N2M0 | AdC |
| 29 | 73 | IIB | T3N0M0 | SqC |  | 29 | 47 | IIIA | T3N2M0 | Other |
| 30 | 71 | IB | T2N0M0 | AdC |  | 30 | 57 | IIIA | T2N2M0 | AdC |
| AdC, adenocarcinoma; SqC, Squamous cell carcinoma; Other, adenosquamous carcinoma. | | | | | | | | | | |
